# Whole Genome Sequencing Reveals Signatures for Artificial Selection for Different Sizes in Japanese Primitive Dog Breeds

**DOI:** 10.3389/fgene.2021.671686

**Published:** 2021-07-14

**Authors:** Guangqi Lyu, Chunyu Feng, Shiyu Zhu, Shuang Ren, Wanyi Dang, David M. Irwin, Zhe Wang, Shuyi Zhang

**Affiliations:** ^1^College of Animal Science and Veterinary Medicine, Shenyang Agricultural University, Shenyang, China; ^2^Department of Laboratory Medicine and Pathobiology, University of Toronto, Toronto, ON, Canada

**Keywords:** selective sweep, body size, dog, pool-seq, genome

## Abstract

Body size is an important trait in companion animals. Recently, a primitive Japanese dog breed, the Shiba Inu, has experienced artificial selection for smaller body size, resulting in the “Mame Shiba Inu” breed. To identify loci and genes that might explain the difference in the body size of these Shiba Inu dogs, we applied whole genome sequencing of pooled samples (pool-seq) on both Shiba Inu and Mame Shiba Inu. We identified a total of 13,618,261 unique SNPs in the genomes of these two breeds of dog. Using selective sweep approaches, including *F*_ST_, *H*_p_ and XP-CLR with sliding windows, we identified a total of 12 genomic windows that show signatures of selection that overlap with nine genes (*PRDM16, ZNF382, ZNF461, ERGIC2, ENSCAFG00000033351, CCDC61, ALDH3A2, ENSCAFG00000011141*, and *ENSCAFG00000018533*). These results provide candidate genes and specific sites that might be associated with body size in dogs. Some of these genes are associated with body size in other mammals, but 8 of the 9 genes are novel candidate genes that need further study.

## Introduction

**FIGURE 1:**
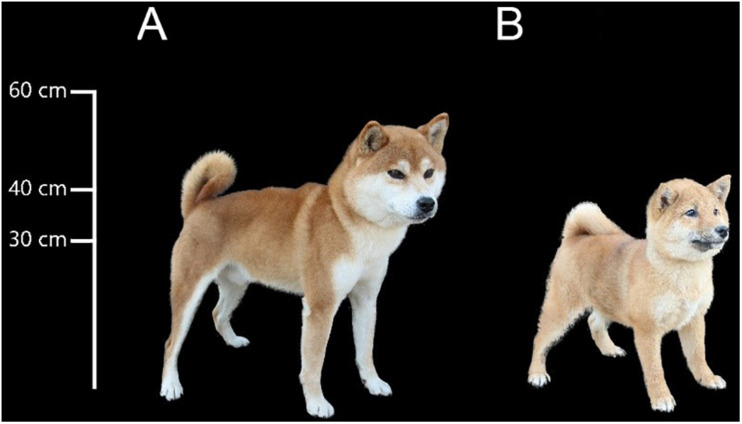
Body sizes of Shiba Inu and Mame Shiba Inu. **(A)** Shiba Inu. **(B)** Mame Shiba Inu.

As our best friend, dogs are crucial to modern human society and are involved in many aspects of our life. During domestication, many traits found in dogs diverged greatly from their ancestors. The Shiba Inu (“inu” means dog in Japanese) is an ancient and native dog breed in Japan, and it is a basal spitz breed (Parker et al., 2017). Shiba Inus typically range in height from 34 to 41 cm (Figure 1A)^1^ although in recent years, artificial selection has been imposed to yield smaller individuals, which are called mini or Mame Shiba Inu (Mame is a Japanese word for “bean,” representing “small”). It is bred only from Shiba Inu to keep “pure ancestry,” and shares some traits with Shiba Inu, such as coat color. The body height of an adult Mame Shiba Inu should be not more than 34 cm (Figure 1B). Although this new breed, the Mame Shiba Inu, has not yet been approved by the American Kennel Club (AKC), it is approved by Kennel Club of Japan and well recognized as a breed in Japan and China.

Regulation of dog body size, or weight, in the dog has been extensively researched, with many studies conducted at the genomic level (Ostrander et al., 2017). Through these works, many genes and loci have been identified as associated with the regulation of body size in the dog. For instance, *IGF1* is a well-studied body size gene. Using a genome-wide scan in Portuguese water dogs, a single *IGF1* SNP haplotype was linked as a major contributor to small body size (Sutter et al., 2007). Consistently, results reported from a study involving 93 dog breeds found that individuals with different *IGF1* SNP and SINE insertion alleles have significant differences in body weight (Rimbault et al., 2013). An *IGF1* SNP was also reported in a study that applied both selective sweep and GWAS analyses in 722 canids, including purebred dogs and wolfs (Plassais et al., 2019). SNPs not only in *IGF1*, but also its receptor, *IGF1R* (e.g., a non-synonymous SNP at chr3: 44,706,389) was found to be associated with reduced size in dogs (Hoopes et al., 2012). GWAS studies have also implicated other genes with SNPs linked to body size. *HMGA2* was reported to be associated with body size where individuals with the ancestral SNP allele in the *HMGA2* 5′UTR have higher body weight than those with the derived allele (Rimbault et al., 2013), as well as another variation between exon 1 and 2 (Chr10: 8351907) was also reported to have an effect on body weight (Plassais et al., 2019). A genomic region within *CDK4* was reported to be associated with size through an across-breed GWAS analysis that involved 8 different dog breeds (Vaysse et al., 2011). With a genome-wide scan on two German shepherd dog families with microsatellite markers identified a contracted DNA repeat in intron 5 of *LHX3* that was associated with dwarfism (Voorbij et al., 2011). A quantitative GWAS was also used on 1,873 dogs, from 158 breeds, which identified an interval on Chromosome X that is upstream of the *ARHGAP36*, *IGSF1*, and *OR5AK2* genes, which was strongly associated with body size (Hayward et al., 2016). Using a GWAS analyses of 690 dogs, three genes (*IRS4*, *IGSF1*, and *ACSL4*) were found to be associated with body weight, where *IRS4* and *IGSF1* are both involved in the GH/IGF1 and thyroid hormonal pathways involved in body size regulation (Plassais et al., 2017).

While many studies have focused on the variation of body size in dogs, it is still possible that other novel genes and loci have been involved in the selection for body size in dogs. The change in body size between Shiba Inu and Mame Shiba Inu is relatively mild, but distinct, making them good source for investigating mechanisms that change body size. In addition, these breeds are highly divergent from the other domesticated dogs. Here we performed whole-genome pool-sequencing (pool-seq) on Shiba Inu and Mame Shiba Inu dogs to try to identify key regions that experienced selection and may regulate their different body sizes.

## Materials and Methods

### Ethics Statement

Owners of the dogs studied here provided informed consent for the use data of their dogs in this scientific research. All procedures used in this study was approved by the Animal Care and Use Committee of Shenyang Agricultural University.

### Sample Preparation and Sequencing

Blood samples from a total of 94 dogs (59 Shiba Inu and 35 Mame Shiba Inu dogs) were collected by venipuncture of their forelimb. For genome sequencing, four pooled samples were generated. The first pooled sample was from 35 Mame Shiba Inu dogs, while the other three were from 29, 22, and 8 Shiba Inu dogs. For each pool, an equivalent volume of blood from each individual of the group was mixed to yield a total volume of 1 mL. Genomic DNA was then extracted from each pool sample using a whole blood genomic DNA extraction kit (TIANGEN, DP318). Genomic DNA were checked for concentration and purity, and then sent to the Beijing Genome Institute (Beijing, China) for library construction (insert size ∼ 300 bp) and genome sequencing. After adapter ligation and DNA cluster preparation, the libraries were then subjected to Illumina HiSeq X Ten for sequencing (150 bp, paired-end). To yield similar sequencing depths for the two dog breeds, the Mame Shiba Inu pool sample was sequenced to 30× depth while each of the three Shiba Inu pool samples were only sequenced to a 10× depth (thus generating 30× for the breed).

### Mapping and SNP Calling

To find loci that might be associated with the body size difference between the two breeds, reads from the three Shiba Inu dog pool-seq samples were merged into one pair of fastq files. Raw reads were processed using the PoolParty pipeline (Micheletti and Narum, 2018). Briefly, raw reads were filtered using BBDuk^2^, with thresholds of 20 for base quality and 25 for minimum read length. Clean reads were mapped to the reference dog genome (assembly CanFam 3.1)^3^ using BWA 0.7.12-r1039 (Li and Durbin, 2009), and the BAM (Binary Alignment Map) files were sorted by coordinate using the Picard tools^4^. To eliminate bias caused by PCR during library preparation, SAMBLASTER (Faust and Hall, 2014) was used to mark duplicates. Subsequently, SAMtools (Li et al., 2009) and BCFtools (Li, 2011) were used to call variants and generate mpileup files, which were used in the downstream analyses. SNPs with minor allele frequency (MAF) < 0.05, depth < 10, quality < 20 and located within 15 bp of an indel were discarded.

### Detection of Selective Sweeps

To identify genomic regions affected by selection, we applied three types of selective sweep analyses, the fixation index (*F*_ST_), the pooled heterozygosity (*H*_p_) and the cross-population composite likelihood ratio test (XP-CLR) approaches. *F*_ST_ in 25-kb non-overlapped sliding windows between the two breeds was calculated using the “fst-sliding.pl” module in Popoolation2 (Kofler et al., 2011), according to the Weir and Cockerham method (Weir and Cockerham, 1984). In the case that we had two populations and their different pooling strategies, the *F*_ST_ estimator is:

(1) $${\hat{F}}_{ST}^{WC}=1-\frac{2\frac{n_{1}n_{2}}{n_{1}+n_{2}}\frac{1}{n_{1}+n_{2}-2}\left[n_{1}{\tilde{p}}_{1}\left(1-{\tilde{p}}_{1}\right)+n_{2}{\tilde{p}}_{2}\left(1-{\tilde{p}}_{2}\right)\right]}{\overset{{\frac{n_{1}n_{2}}{n_{1}+n_{2}}\left({\tilde{p}}_{1}-{\tilde{p}}_{2}\right)}^{2}+\left(2\frac{n_{1}n_{2}}{n_{1}+n_{2}}-1\right)}{\frac{1}{n_{1}+n_{2}-2}\,\left[n_{1}\,{\tilde{p}}_{1}\,\left(1-{\tilde{p}}_{1}\right)+n_{2}\,{\tilde{p}}_{2}\,\left(1-{\tilde{p}}_{2}\right)\right]}}$$

where *n_i_* is the sample size and ${\tilde{p}}_{i}$ is the sample allele frequency of the populations (Bhatia et al., 2013). *H*_p_ and negative Z-transformed Hp (−ZH_p_) of the Mame Shiba Inu were calculated using a custom python3 script from 25-kb non-overlapped sliding windows using the following formulas:

$$\begin{array}{c} Hp=\frac{2\sum n_{MAJ}\sum n_{MIN}}{{\left(\sum n_{MAJ}+\sum n_{MIN}\right)}^{2}}\\ -\text{ZHp}=-\frac{Hp-\mu Hp}{\sigma Hp} \end{array}$$

Z transformation allowed us to compare the two breeds at the same level, since ZH_p_ values indicate the number of standard deviations by which the *H*_p_ value deviates from the mean (Rubin et al., 2010). XP-CLR statistics (Chen et al., 2010) between Mame Shiba Inu and Shiba Inu was calculated using *xpclr*^5^ in 25-kb non-overlapped windows. Windows containing < 10 SNPs were discarded to prevent spurious signals. Windows that were all in the top 1% of the *F*_ST_, -ZH_p_ and normalized XP-CLR score distributions were considered to be candidate selective sweep regions. Genes overlapping with these regions were identified using the ENSEMBL dog genome gene annotations (CanFam 3.1).

## Results

### Sequencing Data, Mapping, and SNPs Calling

In total, we obtained 192.5 Gb of clean sequence data with an average sequencing depth of 39.98× for the two breeds, which was sufficient for the downstream analyses (Table 1). The average coverage against the reference genome was 98.65%, indicating a good quality for the data. We identified 13,618,261 unique variants in the two breeds, of which 11,552,094 (84.8%) were retained after quality control that included 9,242,589 SNPs and 2,309,505 indels. We compared these variants to dbSNP database, 28.22% of these variants matched 58.91% of the records in dbSNP. The Ts/Tv ratios were 1.848 and 1.856 for Shiba Inu and Mame Shiba Inu, while the ratio was 2.09 when computing with dbSNP dataset.

**TABLE 1:** Sequencing statistics of the clean data from the pooled samples.

Sample (individuals)	Bases (G)	Reads (M)	Q20 rate (%)	Q30 rate (%)
Shiba Inu (29)	35.70	238.03	98.16	94.82
Shiba Inu (22)	33.48	223.17	98.39	95.37
Shiba Inu (8)	40.13	267.54	98.43	95.46
Mame Shiba Inu (35)	83.19	554.61	98.45	95.51

### Detection of Selective Sweeps

**FIGURE 2:**
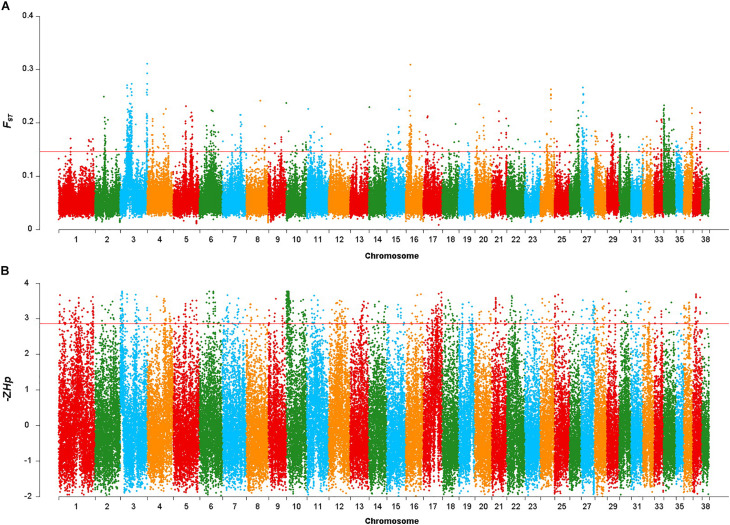
Genomic regions with signatures of selective sweeps identified using 25-kb non-overlapping windowed Fst and Hp approaches. **(A)** Plot of the Fst values in the Shiba Inu vs. Mame Shiba Inu. Red line indicates the significance threshold of the top 1% (Fst = 0.146). **(B)** Plot of the –ZHp values of Mame Shiba Inu. Red line indicates the significance threshold of the top 1% (–ZHp = 2.860).

After discarding windows containing < 10 SNP in the *F*_ST_ sliding window approach, 86,886 windows were retained. The mean *F*_ST_ value was 0.058, with the highest and lowest *F*_ST_ values being 0.311 and 0.008, respectively. A total of 148 gene colocalized in the 869 windows in the top 1% of the *F*_ST_ distribution (*F*_ST_ > 0.146, Figure 2A). Co-localized genes included *FGFRL1, GBX1, IQCA1L, RNF212* and *FAM189A1*, as well as some previously well-studied body size genes such as *IGF1, SMAD2* and *LCORL* (Supplementary Table 1).

Similarly, the mean −ZH_p_ value for the windows was −0.029, with the highest and lowest −ZH_p_ values being 3.767 and −2.019, respectively. We identified 411 genes (Supplementary Table 2) in the 869 windows in the top 1% of the −ZH_p_ distribution of the Mame Shiba Inu (−ZH_p_ > 2.86, Figure 2B). *SLTM* and *SRGAP1* were found in windows with the greatest -ZH_p_ divergence.

**FIGURE 3:**
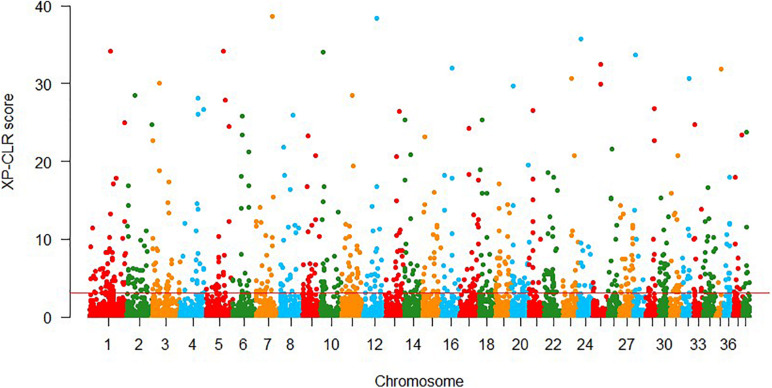
Genomic regions with signatures of selective sweeps identified using 25-kb non-overlapping windowed XP-CLR approaches. Red line indicates the significance threshold of the top 1% (XP-CLR = 3.07).

In XP-CLR approach, 861 putative selection windows were detected using the threshold of 1% cutoff of normalized XP-CLR scores (Figure 3), with normalized XP-CLR scores ranged from 3.07 to 40.58. We identified 442 genes in these windows (Supplementary Table 3), and the windows with highest XP-CLR scores co-localize with genes *ACSBG2*, *UBA5*, and *NPHP3*.

Notably, only 12 windows were located all in the top 1% of the *F*_ST_, -ZH_p_, and XP-CLR score distributions. Within these 12 windows with selective signatures, we identified nine co-localizing genes (*PRDM16, ZNF382, ZNF461, ERGIC2, ENSCAFG00000033351, CCDC61, ALDH3A2, ENSCAFG00000011141*, and *ENSCAFG00000018533*) that are strong candidate selected genes. These windows also included genomic regions, including on Chromosome 3, 8, and 36, which did not overlap with any annotated gene (Table 2).

**TABLE 2:** Regions showing signals of selection with the *F*_ST_, Hp and XP-CLR approaches.

Chr^*a*^	Start^*b*^	End^*c*^	*F*_ST_	Hp^*d*^	−ZHp^*e*^	XP-CLR^*f*^	Gene Symbol^*g*^
1	109,625,000	109,650,000	0.149	0.076	2.873	3.305	*CCDC61*
1	116,300,000	116,325,000	0.149	0.040	3.292	6.477	*ZNF382, ZNF461*
3	52,950,000	52,975,000	0.196	0.008	3.677	15.727	–
5	40,575,000	40,600,000	0.199	0.045	3.232	3.143	*ALDH3A2*
5	42,875,000	42,900,000	0.184	0.060	3.060	9.002	*ENSCAFG00000018533*
5	57,800,000	57,825,000	0.170	0.031	3.406	8.475	*PRDM16*
5	57,825,000	57,850,000	0.211	0.040	3.297	20.152	*PRDM16*
8	34,250,000	34,275,000	0.147	0.044	3.244	24.152	*ENSCAFG00000033351*
8	62,250,000	62,275,000	0.149	0.064	3.015	4.824	–
27	18,625,000	18,650,000	0.217	0.041	3.278	19.421	*ERGIC2*
33	23,675,000	23,700,000	0.169	0.055	3.121	31.624	*ENSCAFG00000011141*
36	7,975,000	8,000,000	0.162	0.040	3.294	25.154	–

**^*a*^Chromosome. ^*b*^Start position of the region. ^*c*^End position of the region. ^*d*^H_p_ value of Mame Shiba Inus. ^*e*^−ZH_p_ value of Mame Shiba Inus. ^*f*^Normalized XP-CLR score of the region. ^*g*^Genes that overlap with the region. Multiple genes are separated with comma. “–” means no overlapping annotated genes in this region.**

## Discussion

Due to human imposed artificial selection, some Shiba Inus now have body sizes that are smaller than their ancestors. In this study, we conducted whole-genome pool sequencing on two breeds of primitive Japanese dogs, Shiba Inu and Mame Shiba Inu, to investigate the molecular mechanisms responsible for the differences in their body size, and to identify candidate genes that may regulate body size in all dogs. In the case that only allele frequencies were needed, pool-seq is well suited for detecting selective sweeps (Boitard et al., 2012). The Ts/Tv ratios were relatively low, probably due to selective forces (Wakeley, 1996). 28.22% of the variants matched dbSNP records, suggesting that there might be breed-specific variants for the two breeds. Using selective sweep analyses, we identified several genomic regions with signatures consistent with selection. When compared to the reference dog genome, we found several genes that are possibly involved in the change in body size of Shiba Inus.

With the *F*_ST_, *H*_p_ and XP-CLR with sliding window approaches, nine genes (*PRDM16, ZNF382, ZNF461, ERGIC2, ENSCAFG00000033351, CCDC61, ALDH3A2, ENSCAFG00000011141*, and *ENSCAFG00000018533*) were found to show selection signatures. Among these genes, *PRDM16* was reported to be associated with growth traits in some species, such as bovine and chicken (Wang et al., 2010; Wu et al., 2021). It is known that *PRDM16* controls a brown fat/skeletal muscle switch (Seale et al., 2008), and it might take a role in the growth of Mame Shiba Inu. Additionally, there are genes identified by two approaches, for instance, *NCAPG* located in a window that had both high *F*_ST_ and low *H*_p_ values (Supplementary Tables 1, 2). *NCAPG-LCORL* region, has previously been reported to be associated with body size or weight in dogs and several other species. The most significant SNP, located on Chromosome 6, identified in a GWAS of 1,781 sheep associated with body weight is in a genomic region that contains the *LAP3*, *NCAPG*, and *LCORL* genes (Al-Mamun et al., 2015). An indel in *LCORL* (Chr3: 105,755,416) and a SNP (Chr3: 105,363,241) 246 kb upstream of *NCAPG-LCORL* were identified to be associated with body size in the Tennessee Walking Horse (Staiger et al., 2016). A SNP (Chr3: 105,547,002) in the *NCAPG-LCORL* region was reported to be associated with the sizes of horses (Makvandi-Nejad et al., 2012). In dogs, a genomic region in *LCORL* (Chr3: 93,933,450-93,944,095) was previously reported to be under selection in dogs (Vaysse et al., 2011). To date, however, there has been no reports linking *PRDM16* or *NCAPG* with body size in dogs. We identified a genomic region (Chr3: 91,275,000–91,300,000) that has both a high *F*_ST_ value (0.212) and low *H*_p_ value (0.045), thus showing a selective signal, between two breeds of Shiba Inu. We also identified selective signals in three well-studied body size genes (*IGF1*, *SMAD2*, and *LCORL*) by the *F*_ST_ approach (Supplementary Table 1), however they did not show extremely low *H*_p_ values, which might indicate that they involved in the change in body size of Shiba Inus during artificial selection, but not in key roles.

Although eight of the nine identified genes (*ZNF382, ZNF461, ERGIC2, ENSCAFG00000033351, ALDH3A2, ENSCAFG00000011141*, and *ENSCAFG00000018533*) and the two genomic regions that do not overlap with any gene have not previously been associated with body size, they all showed signals for selection with the *F*_ST_, *H*_p_, and XP-CLR approaches. We suggest that these genes and genomic regions might possibly have an effect on body size in dogs, they also might be involved in some other traits. More in-depth studies of these genomic regions are required to resolve this question.

In conclusion, using pool-seq data and selective analyses, using the *F*_ST_, *H*_p_, and XP-CLR approaches, we identified nine genes and two genomic regions that experienced selection, including one that were have previously been reported to be associated with body size in mammals (*PRDM16*). Our study identified eight novel genes (*ZNF382, ZNF461, ERGIC2, ENSCAFG00000033351, ALDH3A2, ENSCAFG00000011141*, and *ENSCAFG00000018533*) that need further study. Our results are consistent with previous findings, and provide novel genes and genomic regions that potentially are associated with body size in dogs and other mammals.

## Data Availability Statement

Pool-seq data was submitted to NCBI Sequence Read Archive (accession number PRJNA636107).

## Ethics Statement

The animal study was reviewed and approved by Animal Care and Use Committee of Shenyang Agricultural University. Written informed consent was obtained from the owners for the participation of their animals in this study.

## Author Contributions

GL participated in the design of this study. GL, SR, and WD collected the samples. GL, CF, and SZ performed the experiments. GL, SZ, and ZW analyzed the data. GL, DI, and ZW wrote the manuscript. SZ and ZW conceived, designed the study, and supervised the work. All authors approved the final version of the manuscript.

## Conflict of Interest

The authors declare that the research was conducted in the absence of any commercial or financial relationships that could be construed as a potential conflict of interest.

## Footnotes

1 http://www.akc.org

2 http://sourceforge.net/projects/bbmap/

3 https://www.ncbi.nlm.nih.gov/assembly/GCA_000002285.2

4 http://broadinstitute.github.io/picard/

5 https://github.com/hardingnj/xpclr
